# Pyrroloquinoline Quinone Improved Boar Sperm Quality via Maintaining Mitochondrial Function During Cryopreservation

**DOI:** 10.3390/antiox14010102

**Published:** 2025-01-16

**Authors:** Shanpeng Wang, Qi Wang, Lingjiang Min, Hailiang Cao, Adedeji O. Adetunji, Kaifeng Zhou, Zhendong Zhu

**Affiliations:** 1 College of Animal Science and Technology, Qingdao Agricultural University, Qingdao 266109, China; 2 Qingdao RATO Industrial and Trading Co., Ltd., Qingdao 266000, China; 3 Department of Agriculture, University of Arkansas at Pine Bluff, Pine Bluff, AR 71601, USA; 4 Shandong Provincial Animal Husbandry General Station, Jinan 250022, China

**Keywords:** boar semen, cryopreservation, pyrroloquinoline quinone, sperm quality

## Abstract

Due to oxidative damage and mitochondrial dysfunction, boar semen cryopreservation remains a significant challenge. This study investigates the effects of pyrroloquinoline quinone (PQQ), a mitochondrial-targeted antioxidant, on the post-thaw boar sperm quality during cryopreservation. Boar semen was diluted in a freezing extender containing different concentrations of PQQ (0, 10, 100, 1000, 10,000 nM). After freezing–thawing, the sperm motility, viability, acrosome integrity, mitochondrial activity, adenosine triphosphate (ATP) levels, DNA integrity, malondialdehyde (MDA) levels, reactive oxygen species (ROS) levels, superoxide dismutase (SOD) activity, mitochondrial transcription proteins levels, and fertilization capacity were assessed. The results show that 1000 nM PQQ supplementation to the freezing extender significantly enhanced post-thaw sperm motility, viability, and acrosome integrity compared to the control (*p* < 0.05). Additionally, 1000 nM PQQ increased mitochondrial membrane potential (MMP) and ATP levels, while decreasing MDA and mitochondrial ROS levels, and reducing DNA damage (*p* < 0.05). Furthermore, the levels of mitochondrial-encoded proteins were significantly elevated in the 1000 nM PQQ group compared to the control (*p* < 0.05). Interestingly, sperm in the 1000 nM PQQ group showed a higher binding rate to oviductal epithelial cells and the zona pellucida (ZP), indicating higher fertilization potential. These findings suggest that the use of mitochondria-target antioxidant, PQQ, can improve post-thaw boar sperm quality and fertilization via its capacity to reduce oxidative stress and protect mitochondrial function.

## 1. Introduction

Intensive pig farming primarily relies on liquid-preserved semen for artificial insemination (AI). However, liquid semen faces numerous challenges in transferring genetic material between populations due to its strict temperature requirements during transportation (17–25 °C) and the limited lifespan of sperm [1]. In overcoming these limitations, alternative semen preservation techniques remain a key focus of research. Cryopreservation enables decoupling of semen collection and AI processes in time and space, allowing for the indefinite storage of genetic resources. This technology offers a unique solution for genetic resource management. Although progress has been made in enhancing the boar post-thaw sperm motility and fertilization capacity, the practical application of AI with post-thaw sperm in pigs remains limited [2]. This is because when post-thaw sperm is used for AI in place of fresh semen, lower farrowing rates and live litter sizes are recorded [3]. During the cryopreservation process, sperm plasma membranes are highly susceptible to damage [4]. Membrane damage could impair spermatozoa’s ability to interact effectively with cells in the female genital tract [5], leading to a reduction in fertilization efficiency compared to liquid semen [6]. These issues have hindered the widespread adoption of cryopreservation technology in pig farming.

Boar sperm exhibit heightened sensitivity to cryoinjury, mainly attributed to their high levels of unsaturated fatty acids, necessitating strategies to mitigate its detrimental effects on fertility [7,8]. Furthermore, the limited antioxidant capacity in the cytoplasm of boar sperm [9] makes them highly vulnerable to oxidative damage induced by ROS. During thawing, the mitochondrial electron transport chain (ETC) generates ROS due to electron leakage as the proton gradient across the inner mitochondrial membrane is reestablished [10]. Meanwhile, semen dilution, osmotic fluctuations, and ice crystal formation during cryopreservation further exacerbate ROS production, intensifying oxidative stress in sperm [11,12]. Excessive ROS accumulation impairs mitochondrial function [13], inhibits the mitochondrial ETC, and reduces adenosine triphosphate (ATP) production, subsequently triggering sperm apoptosis and cell death [14,15]. Elevated ROS levels could negatively impact sperm motility, DNA, and membrane integrity. Likewise, it reduces the fertilization capacity of cryopreserved sperm [16,17]. Antioxidants serve as the primary defense components responsible for scavenging ROS [18]. Exogenous antioxidants are usually added to the freezing extender to protect sperm cells from cryopreservation-induced ROS damage. Previous studies showed that supplementation with antioxidants like glutathione, taurine, quercetin, and ascorbic acid could improve the quality of cryopreservation sperm [19,20,21,22].

Pyrroloquinoline quinone (PQQ) is a water-soluble vitamin-like factor with multiple biological activities. Previous studies showed that PQQ plays important roles in inhibiting lipid peroxidation, reducing oxidative damage, and promoting nerve growth factor production [23,24,25]. Additionally, PQQ protects tissue damage caused by hypoxia/ischemia [26] and inflammatory responses [23] and prevents glucocorticoid-induced cataract formation and glutathione depletion [27]. As a redox cofactor, PQQ is an antioxidant and a regulator of various cellular signaling pathways [28]. It has been reported that PQQ could directly scavenge aroxyl and peroxyl radicals to protect cells from oxidative damage [29]. Also, PQQ can promote mitochondrial function [30]. In addition, in Hepa1-6 mouse cells, supplementation with PQQ significantly increased the mitochondrial DNA levels and enhanced the activities of enzymes [31] involved in mitochondrial metabolism. Moreover, supplementation with PQQ can reduce mitochondrial dysfunction by maintaining ATP levels, improving mitochondrial DNA integrity, restoring MMP, and increasing mitochondrial number [32]. Recently, the importance of PQQ in the field of reproduction has gradually gained attention. PQQ improves mitochondrial function by reducing ROS levels, thereby enhancing the proliferation and viability of granulosa cells [33]. Moreover, PQQ alleviates ovarian dysfunction and significantly increases pregnancy rates and litter sizes in mice [34]. Previous studies showed that PQQ significantly improved the quality of chilled ram sperm and post-thaw Holstein bull sperm [35,36]. Furthermore, in our previous study, PQQ supplementation in boar sperm diluted with low-glucose medium preserved high linear motility by reducing ROS levels, stabilizing mitochondrial DNA, and improving mitochondrial activity [37]. However, the effects of PQQ on frozen–thawed boar sperm are limited, and its potential mechanisms remain largely unknown. Therefore, this study aims to evaluate the impact of PQQ on boar sperm quality during cryopreservation, and also to undertake a preliminary exploration of its underlying mechanisms.

## 2. Materials and Methods

### 2.1. Chemicals

All chemicals and reagents were purchased from Sigma-Aldrich (Shanghai, China) unless specified otherwise. The reduced form of PQQ was purchased from MCE (HY-100196, Shanghai, China).

### 2.2. Media Preparation

As described by Zhu et al. (2022) [3], Modena solution was used to prepare the pre-treatment extender and served as the thawing solution. The freezing extender A includes 20% (*v*/*v*) of egg yolk, 80% (*v*/*v*) of 310 mM lactose, and 100 µg/mL polymyxin B, while freezing extender B consists of freezing extender A supplemented with 1.5% (*v*/*v*) Equex-Paste (Minitube, Tiefenbach, Germany) and 4% (*v*/*v*) glycerol.

### 2.3. Animals and Semen Collection

Nine healthy and fertile large white boars sourced from the Jian Aobao Biotechnology Co., Ltd. (Ji'an, China). were utilized in this study. Each boar provided a single ejaculate collected via the gloved-hand technique. The sperm-rich portion of the ejaculate was filtered through a double layer of sterile gauze. Only samples with sperm motility exceeding 90% and an abnormality rate below 15% were used for the study. To minimize variation, the collected ejaculates were pooled before the analysis.

### 2.4. Sperm Processing and Cryopreservation

As described in our previous study [3], after semen collection, the ejaculate was diluted with Modena solution (1:1, *v*/*v*), and cooled to 17 °C. Subsequently, the sperm sample was centrifuged to remove the supernatant. Freezing extender A was used to resuspend the sperm pellets, achieving a final concentration of 2 × 10⁹ sperm/mL. Following this, the sperm suspension was gradually cooled to 4 °C. At this stage, the sperm samples were divided into five groups, each containing different concentrations of PQQ (0, 10, 100, 1000, and 10,000 nM). Next, the sperm samples were mixed with freezing extender B (1:1, *v*/*v*). The prepared samples were transferred into 500 μL plastic straws and moved to a programmable freezer chamber (SIBO Livestock Equipment Co., Ltd., Qingdao, China). The freezing protocol involved the following temperature drop curve: maintaining 4 °C for 100 s, cooling from 4 °C to −1 °C in 90 s, and from −1 °C to −140 °C in 300 s. The plastic straws were subsequently immersed in liquid nitrogen for storage until further analysis. After 7 days of storage, the sperm samples were thawed and incubated for 15 min before further analysis to evaluate the effects of PQQ on the post-thaw boar sperm quality and to choose the optimal concentration of PQQ treatment to elucidate its underlying mechanism of action.

### 2.5. Evaluation of Sperm Motility

As described by our previous study [38], sperm motility parameters were analyzed using a computer-assisted sperm analysis (CASA) system (HT, Beverly, MA, USA). After thawing, the semen sample was loaded onto a Makler chamber (SMI, Haifa, Israel) and evaluated motility in five randomly chosen samples, with each field containing at least 300 sperm.

### 2.6. Thermo-Resistance Test

After thawing, the semen samples were subjected to a thermo-resistance test (TRT) based on the protocol adapted from Schulze et al. (2017) [39]. Semen samples were incubated at 38 °C for up to 300 min and sperm motility parameters were evaluated hourly by a CASA system to monitor changes in sperm motility over time.

### 2.7. Flow Cytometric Evaluation of Sperm Viability and Acrosome Integrity

Sperm viability and acrosome integrity were assessed with FITC-PNA/PI staining. Briefly, 1 mL of diluted semen sample was stained with 0.54 μL of PI (2.4 mM) and 0.6 μL of FITC (1 mg/mL). The sample was mixed and incubated at 38 °C in the dark for 5 min. A flow cytometer (BeamCyte, Changzhou, China) was used to analyze 20,000 events and sperm cells negative for FITC-PNA were classified as having intact acrosome, while those negative for PI were classified as viable sperm cells. The percentage of sperm cells with intact acrosome and viable sperm cells (FITC-PNA-/PI-) was calculated. The analyses were performed in triplicate (n = 3).

### 2.8. Evaluation of Mitochondrial Membrane Potential

Sperm MMP was analyzed with fluorochrome JC-1 dye (65-0851-38, Thermo Fisher Scientific, Boston, MA, USA) as described in our previous study [38]. To establish a negative control, semen containing 10 μM of carbonyl cyanide 3-chlorophenylhydrazone (CCCP) was incubated for 30 min. Subsequently, the sperm sample was stained with 400 μL of JC-1 working solution. After two washes with JC-1 buffer, the sample was resuspended and analyzed by flow cytometry (BeamCyte, Changzhou, China), with 20,000 events recorded. The analyses were performed in triplicate (n = 3).

### 2.9. Measure of Sperm ATP Levels

ATP levels of sperm were quantified using an ATP Assay Kit (ADS-W-A001-96, AIDISHENG, Yancheng, China) following the manufacturer’s protocol. Sperm was disrupted via ultrasound on ice (200 W, 4 s pulses, 12 s intervals, repeated 15 times), and subsequently heated in a boiling water bath for 10 min. Then, 30 μL of lysate sample was incubated with the working solution. Following incubation, a precipitating reagent was added, and the mixture was centrifuged to obtain the supernatant. The supernatant, chromogenic and stop solution were sequentially added to a 96-well plate, and absorbance was read at 636 nm with a microplate reader. The analyses were performed in triplicate (n = 3).

### 2.10. Sperm Oxidative DNA Damage

Oxidized guanine adducts serve as markers of oxidative DNA damage preceding DNA fragmentation. A 100 μL sperm sample was fixed in 4% paraformaldehyde in PBS for 20 min. Afterward, the sample was rinsed with PBS containing 0.5% bovine serum albumin (BSA) and incubated in PBS with 10 mM dithiothreitol for 20 min. The permeabilization step involved a 30 min treatment on ice with 0.1% Triton and 0.1% sodium citrate, followed by another rinse. Subsequently, the sample was incubated with an anti-8-OHdG (bs-1278R, Bioss, Beijing, China) antibody solution. After another wash, a CY3-conjugated secondary antibody was applied, followed by a final wash. After that, the sample was analyzed by a flow cytometer (BeamCyte, Changzhou, China), with a total of 20,000 sperm events recorded for analysis. The analyses were performed in triplicate (n = 3).

### 2.11. Sperm DNA Fragmentation

The terminal deoxynucleotidyl transferase dUTP nick end labeling (TUNEL) assay was used to analyze sperm DNA fragmentation. A 100 μL sample was fixed in 4% paraformaldehyde for 30 min on an orbital shaker. After washing again with PBS, the sample was incubated in PBS with 0.3% Triton X-100 for 5 min. Afterward, 50 μL of TUNEL working solution was applied, followed by a 60 min incubation at 37 °C in the dark. Following two PBS washes, the sperm samples were analyzed by flow cytometry (BeamCyte, Changzhou, China), with 20,000 sperm events recorded for analysis. The analyses were performed in triplicate (n = 3).

### 2.12. Measurement of Sperm MDA Levels

MDA levels in sperm were determined using an MDA Assay Kit (GMS14028, AIDISHENG, Yancheng, China). Sperm samples were lysed on ice, and reagents were added following the manufacturer’s protocol. After heating at 95 °C for 40 min, the mixture was centrifuged, and the supernatant was collected. After heating at 95 °C for 40 min, the mixture was centrifuged, and the supernatant was collected. Absorbance was read at 530 nm using a microplate reader. The analyses were performed in triplicate (n = 3).

### 2.13. Measurement of Sperm SOD Activity

SOD activity in sperm was determined using an SOD Assay Kit (ADS-W-KY011, AIDISHENG, Yancheng, China). Briefly, sperm samples were subjected to ultrasonic disruption (300 W, sonication for 3 s with a 30 s interval, repeated four times). The enzyme working solution was added to the homogenized sperm mixture and incubated at 37 °C for 20 min. Absorbance was measured at 450 nm with a microplate reader. The analyses were performed in triplicate (n = 3).

### 2.14. Detection of Sperm Reactive Oxygen Species

Sperm mitochondrial ROS levels were assessed with the MitoSOX™ Red Assay Kit (M36007, Thermo Fisher Scientific, Boston, MA, USA). Sperm samples were mixed with 400 μL of 5 μM working solution at 37 °C in the dark for 12 min, followed by three washes with PBS. The unstained sperm sample was used as a negative control. A gate was set to distinguish mitochondrial ROS-positive sperms from the negative control. ROS levels were evaluated by flow cytometry (BeamCyte, Changzhou, China), based on 20,000 sperm events. The analyses were performed in triplicate (n = 3).

### 2.15. Western Blotting

Sperm lysates were prepared using RIPA buffer with protease inhibitors via sonication. After lysis, the samples were centrifugated at 12,000× *g* rpm for 15 min. The supernatant was combined with SDS loading buffer and boiled for 10 min. Protein separation was performed by 10% SDS–polyacrylamide gels (SDS-PAGE), followed by proteins being transferred onto a PVDF membrane (Merck Millipore, Darmstadt, Germany). Non-specific binding was blocked using 5% (*m*/*v*) BSA diluted in TBST, and the membranes were incubated with primary antibodies targeting MT-ND1 (A17967, ABclonal, Wuhan, China) and MT-ND6 (A17991, ABclonal, Wuhan, China). The membranes were incubated with an HRP-conjugated secondary antibody (A0208, Beyotime, Shanghai, China) for 1 h. The blots were visualized with a gel imaging analyzer. Quantification of the results was performed using ImageJ software Version 1.54m (NIH, Bethesda, MD, USA), and the relative intensity of the target proteins was normalized using α-tubulin. The analyses were performed in triplicate (n = 3).

### 2.16. The Ability of Frozen–Thawed Sperm to Bind to the Explants Was Evaluated

As described in a previous study [38], the relative fertility of sperm was evaluated based on their binding capacity to oviduct explants. Oviductal tissues were collected from healthy sows. Explants were prepared from the oviductal isthmus and subsequently maintained in Tyrode’s medium. Only explants with intact ciliated structures were used for experiments. Hoechst 33342-stained sperm were incubated for 30 min. Explants (4 per sow) were placed individually into pre-warmed Tyrode’s medium (5% CO_2_, 38 °C, 100% humidity) in 24-well plates and co-incubated with sperm (2 × 10^5^) for 45 min. After washing to remove loosely attached sperm, explants were mounted on glass slides with Tyrode’s medium and sealed with a coverslip. To minimize the edge effects from tissue preparation, the outermost layer of the explant was excluded, and three regions for analysis were randomly chosen within the remaining explant surface area. Images of different focal planes were combined to quantify sperm binding. Explant surface area and sperm count were determined to evaluate binding density.

The average sperm bound per mm^2^ was defined as the binding index (BI), and it is calculated as shown in Equation (1):

(1) BI=(NI1+NI2+NI3)/(AI1+AI2+AI3)

where Al1, Al2, and Al3 represent the areas of three different regions, while Nl1, Nl2, and Nl3 represent the number of sperm bound to each of these regions.

### 2.17. Sperm–Zona Pellucida Binding Capacity

Ovaries were collected from pigs at the slaughterhouse, and 3–8 mm follicles were aspirated using a 3 mL plastic syringe to obtain cumulus–oocyte complexes. According to a previous study [40], the cumulus–oocyte complexes were incubated in PBS containing 0.1% hyaluronidase (*v*:*v*) and slowly vortexed for 5 min to remove the cumulus cells. The zona pellucida (ZP) was prepared from oocytes using a microinjection system (Biocompare, San Francisco, CA, USA). The ZP was placed in a 1000 μL culture medium droplet. Based on our prior research [38], thawed sperm were incubated in a capacitation medium (95 mM NaCl, 4.8 mM KCl, 1.2 mM KH_2_PO_4_, 5.55 mM glucose, 25 mM NaHCO_3_, 2 mM CaCl_2_, 2 mM sodium pyruvate, and 0.4% BSA). Then, 50 μL of capacitated sperm suspension (at a final concentration of 3 × 10^6^ sperm/mL) was added to each PBS droplet containing five ZP, with five droplets used per group (total 25 ZPs). The droplets were incubated for 15 min at 38 °C in a 5% CO_2_. After incubation, the sperm–ZP complexes were gently washed with PBS to remove loosely attached sperm. The sperm attached to the ZP were separated by repeated aspiration using a pipette, followed by counting under a microscope.

### 2.18. Statistical Analysis

Statistical analyses were conducted using IBM SPSS Statistics 27 (IBM Corp., Armonk, NY, USA). Normality and homogeneity of variance tests were conducted before proceeding with statistical analysis. The data were transformed by arcsine square root transformation when necessary. One-way analysis of variance (ANOVA) was applied to compare basic sperm parameters, and pairwise comparisons were made using independent *t*-tests. All data were expressed as mean ± standard deviation (SD). Graphs were created using GraphPad Prism 8.0 (GraphPad Software Inc., La Jolla, CA, USA). Statistical significance was defined as *p* < 0.05.

## 3. Results

### 3.1. PQQ Improved the Frozen–Thawed Boar Sperm Motility, Viability and Acrosome Integrity

As shown in Table 1, supplementation of PQQ to the freezing extender significantly improved the post-thaw boar sperm total motility and progressive motility compared to the control (*p* < 0.05). Specifically, sperm samples supplemented with 1000 nM PQQ exhibited a 23.7% increase in total motility (72.3 ± 2.5% vs. 48.6 ± 5.9%) and a 17.8% increase in progressive motility (34.1 ± 1.7% vs. 16.3 ± 3.1%) when compared to the control group. Moreover, PQQ supplementation significantly improved the post-thaw sperm curvilinear velocity (VCL), straight-line velocity (VSL), straightness (STR), average path velocity (VAP), beat-cross frequency (BCF), and linearity (LIN)compared to the control group (*p* < 0.05). However, there is no significant difference between PQQ treatment and the control for post-thaw sperm ALH and WOB (*p* > 0.05).

**Figure 1:**
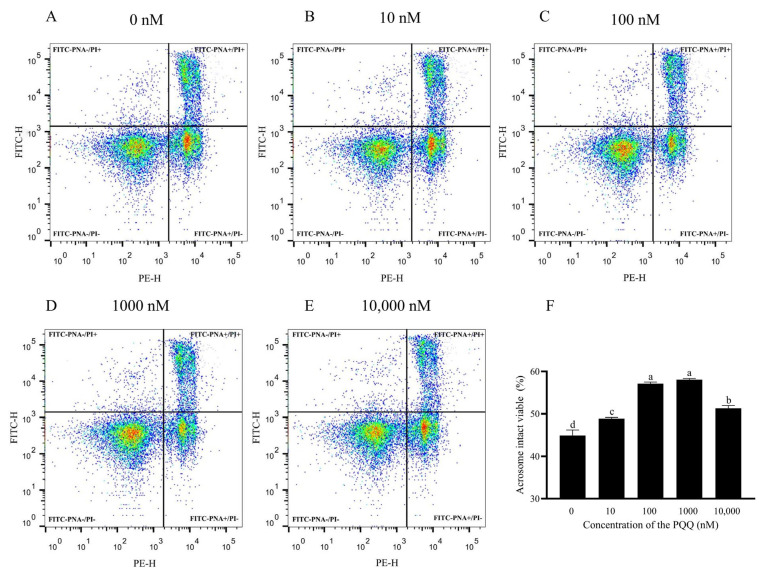
Effect of PQQ added to the freezing extender on post-thaw boar sperm viability and acrosome integrity. (**A**–**E**) Flow cytometry analysis of post-thaw boar sperm viability and acrosome integrity; (**F**) acrosome intact viable sperm (FITC-PNA-/PI-). Statistical analysis was performed using one-way ANOVA followed by Tukey’s post hoc test. Statistical analysis was performed using one-way ANOVA followed by Tukey’s post hoc test. Data are presented as means ± standard deviation, n = 3, with different letters representing significant differences (*p* < 0.05).

In terms of boar post-thaw sperm with acrosome intact and viability, as shown in Figure 1A–F and Figure S1, PQQ supplementation from 100 nM to 1000 nM significantly elevated the percentage of acrosome intact viable sperm (FITC-PNA-/PI-) compared to the control (*p* < 0.05); however, the percentage of acrosome intact viable sperm in the 10 nM PQQ group was similar to that of the control (*p* > 0.05).

### 3.2. PQQ Enhanced the Thermo-Resistance of Frozen–Thawed Sperm

As shown in Table 2, adding PQQ influenced sperm total motility, progressive motility, VCL, VSL, VAP, and ALH following TRT. Sperm samples supplemented with 1000 nM PQQ showed significantly higher values for total motility, progressive motility, VCL, VSL, VAP, and ALH, compared to the other groups (*p* < 0.05). Specifically, after one hour of incubation, sperm total motility in the 1000 nM PQQ group was 24% higher than the control group (71.5 ± 3.5% vs. 47.5 ± 3.9%), while progressive motility was 12.8% higher (29.8 ± 3.0% vs. 17.0 ± 2.8%). After five hours of incubation, the total motility and progressive motility in the 1000 nM PQQ group were respectively 34.6% (71.3 ± 4.3% vs. 36.7 ± 8.8%) and 14.8% (26.5 ± 1.4% vs. 11.7 ± 2.7%) higher than those in the control. Moreover, sperm in the 10,000 nM PQQ group exhibited significantly lower values for total motility, progressive motility, VCL, VSL, VAP, and ALH when compared to those in the 100 nM and 1000 nM PQQ groups (*p* < 0.05).

### 3.3. PQQ Increased the Frozen–Thawed Mitochondrial Membrane Potential and ATP Levels

**Figure 2:**
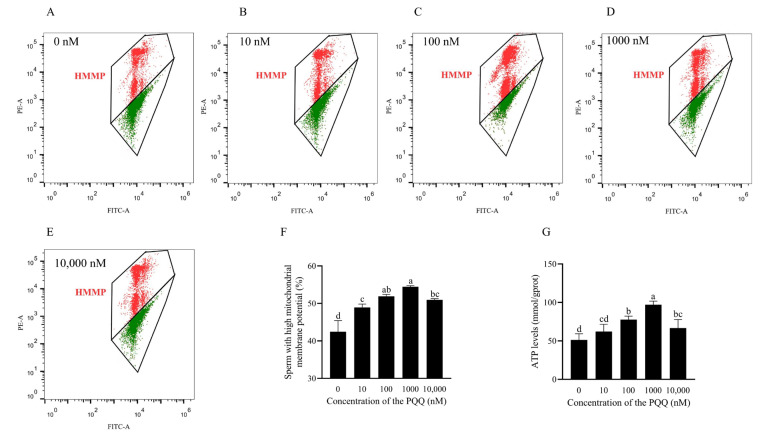
Effects of PQQ added to the freezing extender on post-thaw boar sperm MMP. (**A**–**E**) Flow cytometry evaluation of sperm MMP. Sperm with HMMP are represented by the red region; (**F**) sperm MMP; (**G**) ATP levels. Statistical analysis was performed using one-way ANOVA followed by Tukey’s post hoc test. Data are presented as means ± standard deviation, n = 3, with different letters representing significant differences (*p* < 0.05). HMMP: high mitochondrial membrane potential.

As shown in Figure 2A–F and Figure S2, PQQ supplementation in the freezing extender significantly improved sperm MMP compared to the control (*p* < 0.05), with the 1000 nM PQQ group having the highest values. However, the 10,000 nM PQQ group exhibited a significant decrease in MMP compared to the 1000 nM PQQ group (*p* < 0.05). In addition, PQQ supplementation also significantly improved post-thaw sperm ATP levels (*p* < 0.05; Figure 2G). Moreover, the 1000 nM PQQ-treated group had the highest ATP levels compared to the other group.

### 3.4. PQQ Reduced DNA Oxidative Damage in the Frozen–Thawed Sperm

**Figure 3:**
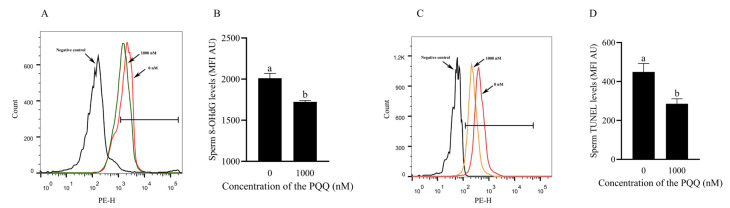
Effects of PQQ added to the freezing extender on post-thaw boar sperm DNA integrity. (**A**,**B**) 8-OHdG levels in sperm were analyzed by measuring the MFI of stained sperm. Higher MFI indicates elevated 8-OHdG levels; (**C**,**D**) TUNEL levels in sperm were assessed by MFI from stained sperm. Higher MFI corresponds to increased TUNEL levels. MFI: mean fluorescence intensity; AU: arbitrary units. Statistical analysis was performed using an independent two-tailed *t*-test. Data are presented as means ± standard deviation, n = 3, with different letters representing significant differences (*p* < 0.05).

Sperm DNA oxidative damage has potentially harmful effects on fertility. To investigate whether PQQ can alleviate oxidative DNA damage in boar sperm, we evaluated TUNEL-stained sperm samples and the oxidative DNA damage marker 8-hydroxy-2’-deoxyguanosine (8-OHdG) using flow cytometry. Figure 3A–D demonstrates that supplementation with 1000 nM PQQ to the freezing extender significantly reduced post-thaw sperm DNA oxidative damage compared to the control group (*p* < 0.05).

### 3.5. PQQ Reduced Oxidative Stress Levels in Frozen–Thawed Sperm

**Figure 4:**
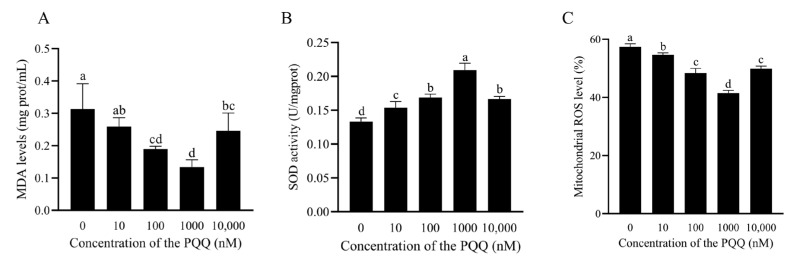
Effects of PQQ added to the freezing extender on post-thaw boar sperm MDA levels (**A**), SOD activity (**B**), and mitochondrial ROS levels (**C**). Statistical analysis was performed using one-way ANOVA followed by Tukey’s post hoc test. MDA: malondialdehyde; SOD: superoxide dismutase; ROS: reactive oxygen species. Data are presented as means ± standard deviation, n = 3, with different letters representing significant differences (*p* < 0.05).

As shown in Figure 4A, MDA levels were significantly lower in the 100 nM and 1000 nM PQQ groups compared to the control group (*p* < 0.05). In contrast, the addition of a higher concentration of PQQ (10,000 nM) to the freezing extender resulted in a significant increase in MDA levels compared to the 100 nM and 1000 nM groups (*p* < 0.05). Additionally, the 1000 nM PQQ treatment exhibited the highest SOD activity in post-thaw sperm (*p* < 0.05; Figure 4B). As shown in Figure 4C and Figure S3, the addition of PQQ from 10 to 10,000 nM to the freezing medium also significantly reduced mitochondrial ROS levels compared to the control group (*p* < 0.05), and the 1000 nM PQQ treatment showed the lowest mitochondrial ROS levels among all the treatment.

### 3.6. PQQ Maintained Boar Sperm Mitochondrial Proteins Levels

**Figure 5:**
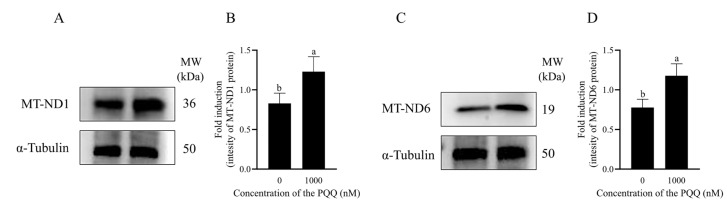
Effects of PQQ added to the freezing extender on post-thaw boar sperm mitochondria-encoded proteins. (**A**,**C**) The Western blotting results illustrate the levels of MT-ND1, MT-ND6, and α-tubulin proteins in boar sperm samples; (**B**,**D**) using α-tubulin as the reference protein, the relative levels of MT-ND1 and MT-ND6 were quantitatively determined. Statistical analysis was performed using an independent two-tailed *t*-test. Data are presented as means ± standard deviation, n = 3, with different letters representing significant differences (*p* < 0.05).

Western blot analysis was employed to examine the effect of PQQ on the levels of mitochondrial-encoded protein. As demonstrated in Figure 5A–D and Figure S4, the addition of PQQ to the freezing extender significantly increased the total levels of MT-ND1 and MT-ND6 compared to the control group (*p* < 0.05).

### 3.7. PQQ Improved the Binding Capacity of Frozen–Thawed Sperm to Explants and Zona Pellucida

**Figure 6:**
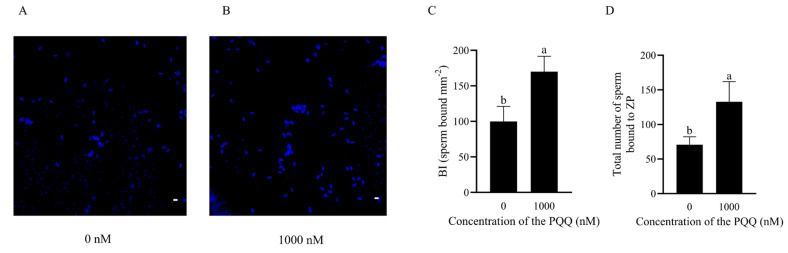
Effects of PQQ added to the freezing extender on the post-thaw binding capacity of boar sperm to oviductal explants. (**A**,**B**) Example image showing sperm bound to the oviduct explant surface (bar = 10 μm); (**C**) sperm BI, n = 4; (**D**) total number of sperm bound to zona pellucida (ZP), n = 25. Statistical analysis was performed using an independent two-tailed *t*-test. Data are presented as means ± standard deviation, with different letters representing significant difference (*p* < 0.05).

To explore the effect of PQQ in the freezing extender on post-thaw boar sperm fertilization capacity, Hoechst 33342 staining was performed, and BI was used to measure sperm reservoir formation ability in the oviduct. As illustrated in Figure 6A–C, sperm BI in the 1000 nM PQQ group was significantly increased compared to the control group (*p* < 0.05), suggesting that PQQ supplementation enhances reservoir-forming potential of frozen–thawed sperm. Furthermore, the addition of 1000 nM PQQ also significantly increased the number of sperm bound to the ZP compared to the control group (Figure 6D and Figure S5, *p* < 0.05).

## 4. Discussion

Optimization of cryopreservation techniques to preserve sperm quality is critical for advancing applications in animal reproduction and biotechnology; however, it remains a complex challenge. Due to the limited cytoplasmic space in sperm, they typically lack sufficient endogenous antioxidants [9], making them particularly vulnerable to oxidative damage during cryopreservation. Moreover, antioxidants in seminal plasma help protect sperm from oxidative damage [41]. During boar sperm cryopreservation, removing seminal plasma from semen increases the vulnerability of sperm to oxidative damage [42]. While conventional antioxidants have become a common strategy to alleviate oxidative stress [43], targeted delivery of antioxidants to ROS generation sites offers greater advantages in reducing oxidative stress and enhancing semen cryopreservation outcomes compared to traditional antioxidants [37,44,45,46]. As a novel mitochondrial-targeted antioxidant, PQQ has shown the potential to improve sperm quality during preservation, but its effects on boar sperm remain unclear. In this study, we observed that adding 1000 nM PQQ to the freezing extender significantly increased sperm motility, and improved motility parameters, viability, and acrosome integrity. This suggests that PQQ can alleviate damage during cryopreservation, thereby improving sperm quality. Similar effects were reported in studies on Holstein bull sperm [36]. However, we also observed that higher concentrations of PQQ (10,000 nM) resulted in a reduction in sperm quality. This phenomenon may be related to the concentration of PQQ being significantly higher than those typically observed in normal blood or tissues (3–40 nM) [47,48]. Furthermore, PQQ plays a crucial role in enhancing mitochondrial function by increasing the activity of NAD^+^-dependent deacetylases sirtuin 1 (SIRT1) and sirtuin 3 (SIRT3). While moderate activation of SIRT1 and SIRT3 is essential for maintaining cellular function, excessive PQQ may adversely affect sperm viability by overstimulating the activity of SIRT1 and SIRT3. The appropriate concentration of PQQ in freezing extenders varies across studies, likely due to differences in animal species, freezing extender composition, and semen handling procedures.

Post-thaw sperm motility is a reliable indicator of the effectiveness of cryopreservation and a predictor of sperm fertility [49]. During the freezing process, the functional integrity of sperm is challenging to assess using conventional motility tests, making methods like the TRT invaluable for detecting subtle sperm damage [50]. In addition, to achieve successful fertilization, viable and non-capacitated sperm is required to bind with epithelial cells of the oviduct [51]. In this study, sperm treated with 1000 nM PQQ exhibited improved motility in the TRT and higher binding rates to both explants and the ZP, emphasizing PQQ’s role in preserving sperm functional integrity and enhancing fertilization potential after thawing.

Boar sperm membranes are highly enriched in polyunsaturated fatty acids, rendering them particularly sensitive to ROS-induced oxidative stress [52]. Accumulation of ROS can lead to a depletion of intracellular ATP, which is associated with reduced motility and male infertility [53,54]. In the present study, the addition of 1000 nM PQQ to the freezing extender significantly reduced mitochondrial ROS levels. This suggests that PQQ mitigates oxidative damage during cryopreservation, thereby enhancing sperm quality. However, when a higher concentration of PQQ (10,000 nM) was used, we observed a significant increase in mitochondrial ROS levels. This increase in mitochondrial ROS suggested that excessive PQQ may overwhelm the antioxidant defense system, leading to mitochondrial stress and oxidative damage. ROS-induced oxidative stress can cause damage to cellular proteins, leading to their degradation and functional loss [55]. Mitochondria are the main source of ROS, with Complex I and Complex III acting as key sites of ROS production in the ETC [37]. As subunits of Complex I situated near ROS generation sites [56], MT-ND1 and MT-ND6 are particularly susceptible to ROS-induced oxidative damage. In this study, PQQ protects these proteins from oxidative damage, thereby supporting the functionality of the ETC and ATP production. These findings are consistent with previous studies, which have shown that the preserving of mitochondrial proteins can enhance mitochondrial function and energy production in sperm [37]. MMP is a critical indicator of overall mitochondrial health and plays a crucial role in ATP generation [57]. MMP is associated with the proton gradient generated during electron transport through the ETC [58]. A decrease in membrane potential is indicative of mitochondrial dysfunction and is associated with reduced ATP production [59]. Mitochondrial dysfunction and insufficient energy production are key contributors to reduced sperm motility [60]. In this study, a significant increase in MMP and ATP content in the 1000 nM PQQ group was observed, suggesting that PQQ enhances mitochondrial function. This, in turn, supports improved sperm motility and overall sperm quality. However, in the 10,000 nM PQQ group, we observed a significant decrease in MMP and ATP levels, which coincided with an increase in ROS, indicating that excessive PQQ caused mitochondrial dysfunction and impaired sperm motility.

MDA is widely recognized as a marker of lipid peroxidation and oxidative stress [61]. In addition, SOD is a critical antioxidant enzyme in male germ cells that neutralizes ROS and prevents cellular damage induced by oxidative stress [62]. In this study, 1000 nM PQQ reduced MDA levels and increased SOD activity, suggesting that PQQ mitigates oxidative stress during cryopreservation. In contrast, in the 10,000 nM PQQ group, elevated MDA and reduced SOD activity indicated that excessive PQQ may induce mitochondrial ROS, leading to oxidative damage and decreased sperm quality. Sperm DNA integrity is closely associated with male fertility potential [63], as DNA damage can lead to poor fertilization outcomes and affect litter size [64]. Evaluating sperm DNA integrity serves as a valuable complementary tool in semen analysis. The process of DNA fragmentation in sperm is primarily driven by oxidative stress, wherein ROS induces single-strand breaks in DNA. These breaks are typically the result of an imbalance between ROS production and the antioxidant defense system. ROS can also directly interact with nitrogenous bases in DNA, leading to oxidative DNA damage, such as the formation of 8-OHdG. 8-OHdG is a common marker for oxidative DNA damage and is often excised during repair processes, contributing to the formation of DNA strand breaks [65]. TUNEL assays were developed to analyze DNA fragmentation in sperm. The TUNEL assay and 8-OHdG measurement revealed that 1000 nM PQQ reduced both 8-OHdG levels and TUNEL-positive sperm, improving DNA integrity by mitigating oxidative DNA damage.

**Figure 7:**
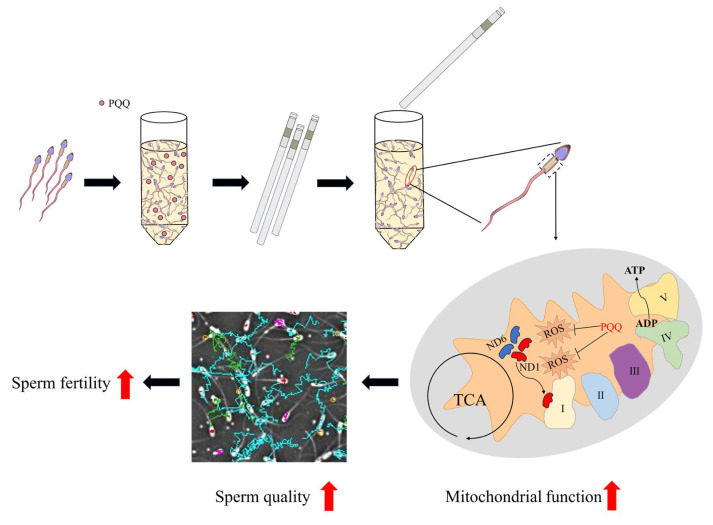
The mechanism of PQQ supplementation in the freezing extender in enhancing post-thaw boar sperm quality. As a mitochondrial-targeted antioxidant, PQQ lowers ROS levels produced during freezing and thawing. By alleviating oxidative stress, PQQ helps protect mitochondrial function, stabilizes ETC proteins MT-ND1 and MT-ND6, and maintains ATP production, improving post-thaw sperm motility and fertilization.

In conclusion, as shown in Figure 7, ROS, a byproduct of mitochondrial ATP production, is generated during freezing and thawing processes. Adding the mitochondrial-targeted antioxidant PQQ to the freezing extender can reduce ROS levels and protect DNA integrity and ETC proteins such as MT-ND1 and MT-ND6 involved in maintaining ATP generation in the mitochondria. Overall, the supplementation of PQQ to the freezing extender enhances boar post-thaw sperm motility and fertilization potential. These findings provide a straightforward strategy for enhancing the application of frozen semen in swine AI. However, it should be noted that the effects of PQQ-treated sperm following in vivo insemination have not been evaluated, and further studies are needed to confirm its in vivo impact.

## Figures and Tables

**Table 1:** Effects of PQQ added to the freezing extender on post-thaw boar sperm motility parameters.

Parameters	0 nM	10 nM	100 nM	1000 nM	10,000 nM
Total motility (%)	48.6 ± 5.9 ^d^	51.1 ± 4.8 ^d^	68.2 ± 2.2 ^b^	72.3 ± 2.5 ^a^	60.9 ± 4.7 ^c^
Progressive motility (%)	16.3 ± 3.1 ^d^	21.7 ± 1.3 ^c^	31.9 ± 1.7 ^a^	34.1 ± 1.7 ^a^	26.2 ± 3.5 ^b^
VCL (μm/s)	97.5 ± 2.8 ^c^	103.4 ± 5.6 ^b^	115.0 ± 2.4 ^a^	112.2 ± 2.3 ^a^	102.9 ± 2.2 ^b^
VSL (μm/s)	27.3 ± 1.0 ^c^	33.0 ± 2.0 ^b^	36.0 ± 1.4 ^a^	36.2 ± 1.2 ^a^	33.2 ± 0.9 ^b^
VAP (μm/s)	48.5 ± 1.5 ^c^	51.8 ± 3.0 ^b^	57.5 ± 1.4 ^a^	57.1 ± 1.7 ^a^	51.8 ± 1.1 ^b^
STR (%)	54.8 ± 1.0 ^c^	61.0 ± 2.2 ^a^	59.2 ± 1.6 ^b^	60.3 ± 1.6 ^ab^	60.5 ± 1.3 ^ab^
LIN (%)	28.8 ± 1.0 ^c^	31.7 ± 2.0 ^a^	30.3 ± 1.0 ^b^	31.4 ± 0.9 ^ab^	31.6 ± 0.9 ^a^
WOB (%)	50.9 ± 1.0	50.5 ± 1.5	49.7 ± 0.9	50.5 ± 0.7	50.0 ± 0.8
ALH (μm)	8.2 ± 0.3	8.7 ± 0.3	8.5 ± 0.2	8.3 ± 0.3	8.6 ± 0.2
BCF (Hz)	34.4 ± 1.8 ^d^	37.2 ± 1.3 ^c^	39.6 ± 1.0 ^b^	39.0 ± 0.8 ^b^	42.6 ± 1.2 ^a^

Statistical analysis was performed using one-way ANOVA followed by Tukey’s post hoc test, n = 3. Data are presented as means ± standard deviation, with different letters representing significant differences (*p* < 0.05). VCL, curvilinear velocity; VSL, straight-line velocity; VAP, average path velocity; BCF, beat-cross frequency; ALH, lateral head; STR, straightness (VSL/VAP); LIN, linearity (VSL/VCL); WOB, wobble (VAP/VCL).

**Table 2:** Effects of PQQ added to the freezing extender on post-thaw boar sperm thermo-resistance.

Parameters	Time (h)	0 nM	10 nM	100 nM	1000 nM	10,000 nM
Total motility (%)	1	47.5 ± 3.9 ^e^	51.7 ± 3.9 ^d^	66.5 ± 4.6 ^b^	71.5 ± 3.5 ^a^	56.0 ± 3.2 ^c^
	2	47.7 ± 4.2 ^d^	51.7 ± 3.7 ^c^	65.9 ± 2.2 ^b^	72.0 ± 4.8 ^a^	52.3 ± 3.4 ^c^
	3	45.3 ± 3.3 ^d^	46.0 ± 4.5 ^d^	61.5 ± 4.5 ^b^	71.2 ± 4.2 ^a^	52.0 ± 3.4 ^c^
	4	40.6 ± 2.7 ^d^	45.9 ± 2.7 ^c^	61.7 ± 4.6 ^b^	71.1 ± 4.7 ^a^	47.6 ± 4.1 ^c^
	5	36.7 ± 8.8 ^d^	44.5 ± 3.9 ^c^	60.2 ± 5.2 ^b^	71.3 ± 4.3 ^a^	38.0 ± 5.2 ^c d^
Progressive motility (%)	1	17.0 ± 2.8 ^d^	22.9 ± 2.4 ^c^	27.0 ± 3.0 ^b^	29.8 ± 3.0 ^a^	21.4 ± 2.8 ^c^
	2	15.0 ± 1.9 ^d^	16.0 ± 3.1 ^d^	26.5 ± 2.7 ^b^	30.4 ± 4.2 ^a^	22.4 ± 2.4 ^c^
	3	13.1 ± 2.6 ^d^	14.5 ± 3.0 ^d^	21.7 ± 2.8 ^b^	31.9 ± 6.4 ^a^	17.8 ± 1.9 ^c^
	4	12.4 ± 1.3 ^e^	15.5 ± 1.9 ^d^	22.2 ± 3.3 ^b^	26.7 ± 2.1 ^a^	18.0 ± 2.2 ^c^
	5	11.7 ± 2.7 ^d^	16.0 ± 2.5 ^c^	21.5 ± 3.9 ^b^	26.5 ± 1.4 ^a^	11.9 ± 2.4 ^d^
VCL (μm/s)	1	95.5 ± 6.9 ^b^	114.1 ± 3.1 ^a^	111.9 ± 4.2 ^a^	111.3 ± 5.1 ^a^	99.3 ± 4.2 ^b^
	2	90.5 ± 3.1 ^b^	106.8 ± 3.6 ^a^	109.6 ± 5.7 ^a^	110.9 ± 7.0 ^a^	94.5 ± 4.2 ^b^
	3	94.1 ± 4.2 ^c^	107.9 ± 7.0 ^ab^	105.8 ± 4.7 ^ab^	111.7 ± 7.1 ^a^	102.2 ± 1.8 ^b^
	4	92.1 ± 5.8 ^c^	91.5 ± 3.3 ^c^	99.2 ± 5.7 ^b^	112.4 ± 9.8 ^a^	97.8 ± 3.0 ^b^
	5	91.6 ± 4.3 ^d^	97.1 ± 4.5 ^c^	106.4 ± 3.9 ^b^	116.1 ± 2.6 ^a^	97.2 ± 6.4 ^c^
VSL (μm/s)	1	28.9 ± 2.3 ^b^	33.6 ± 1.8 ^a^	32.3 ± 1.0 ^a^	33.1 ± 1.6 ^a^	29.8 ± 1.9 ^b^
	2	24.9 ± 1.4 ^c^	31.1 ± 1.6 ^a^	31.9 ± 2.1 ^a^	31.7 ± 2.0 ^a^	27.7 ± 1.4 ^b^
	3	24.8 ± 1.2 ^c^	29.5 ± 1.7 ^b^	30.1 ± 1.4 ^ab^	31.5 ± 2.7 ^a^	29.0 ± 0.9 ^b^
	4	25.0 ± 1.8 ^c^	25.5 ± 1.7 ^bc^	27.2 ± 1.8 ^b^	32.8 ± 3.1 ^a^	27.1 ± 1.6 ^b^
	5	25.5 ± 1.4 ^d^	27.7 ± 1.4 ^c^	29.9 ± 1.4 ^b^	32.6 ± 1.2 ^a^	28.4 ± 1.9 ^c^
VAP (μm/s)	1	47.1 ± 2.9 ^c^	56.6 ± 2.2 ^a^	55.4 ± 1.8 ^a^	55.7 ± 2.3 ^a^	49.1 ± 2.2 ^b^
	2	42.0 ± 2.4 ^c^	52.1 ± 1.9 ^a^	54.2 ± 3.4 ^a^	54.1 ± 3.2 ^a^	46.0 ± 2.0 ^b^
	3	42.1 ± 2.3 ^c^	51.3 ± 2.5 ^ab^	52.6 ± 2.3 ^ab^	54.8 ± 5.5 ^a^	49.0 ± 1.3 ^b^
	4	41.8 ± 2.9 ^c^	41.8 ± 2.1 ^c^	47.1 ± 2.8 ^b^	55.5 ± 4.9 ^a^	45.6 ± 1.9 ^b^
	5	43.5 ± 1.9 ^d^	46.6 ± 2.2 ^c^	50.9 ± 2.3 ^b^	55.1 ± 1.9 ^a^	46.9 ± 2.8 ^c^
STR (%)	1	59.3 ± 3.1 ^a^	57.5 ± 2.2 ^ab^	55.3 ± 1.5 ^c^	57.2 ± 1.3 ^b^	57.8 ± 1.9 ^ab^
	2	57.9 ± 2.0 ^ab^	57.8 ± 1.5 ^ab^	56.7 ± 1.8 ^bc^	56.0 ± 1.8 ^c^	58.8 ± 1.7 ^a^
	3	56.4 ± 1.9 ^ab^	55.1 ± 1.2 ^b^	56.5 ± 1.8 ^ab^	57.4 ± 1.6 ^a^	57.2 ± 1.8 ^ab^
	4	58.3 ± 1.6 ^a^	58.6 ± 2 ^a^	55.2 ± 1.7 ^c^	56.9 ± 0.9 ^b^	56.9 ± 1.2 ^b^
	5	56.6 ± 2.5 ^b^	56.5 ± 2.1 ^b^	56 ± 1.4 ^b^	57.5 ± 1.1 ^b^	59.7 ± 1.9 ^a^
LIN (%)	1	30.2 ± 1.8 ^a^	29.2 ± 1.4 ^ab^	28.1 ± 1.1 ^b^	29.3 ± 0.6 ^ab^	29.4 ± 1.3 ^ab^
	2	27.5 ± 1.1 ^c^	28.3 ± 0.9 ^bc^	28.4 ± 1.0 ^b^	28.0 ± 1.2 ^bc^	29.5 ± 1.1 ^a^
	3	26.5 ± 1.2 ^b^	26.9 ± 0.6 ^b^	29.2 ± 2.0 ^a^	28.7 ± 1.2 ^a^	28.1 ± 1.1 ^ab^
	4	26.9 ± 1.2 ^b^	27.2 ± 1.1 ^b^	27.0 ± 1.1 ^b^	28.5 ± 0.6 ^a^	27.2 ± 0.9 ^b^
	5	28.4 ± 2.2 ^b^	28.3 ± 1.3 ^b^	27.3 ± 0.6 ^b^	27.9 ± 0.8 ^b^	29.8 ± 1.6 ^a^
WOB (%)	1	49.2 ± 1.4	49.0 ± 1.0	48.9 ± 0.5	49.5 ± 0.4	49.1 ± 1.3
	2	46.5 ± 1.4 ^c^	47.7 ± 0.5 ^b^	48.7 ± 0.8 ^a^	48.5 ± 1.2 ^ab^	48.7 ± 1.1 ^a^
	3	44.9 ± 1.3 ^b^	47.3 ± 1.3 ^a^	49.4 ± 2.7 ^a^	48.6 ± 1.9 ^a^	47.6 ± 0.8 ^a^
	4	45.1 ± 1.0 ^d^	45.1 ± 1.2 ^d^	47.3 ± 0.9 ^b^	48.6 ± 0.5 ^a^	46.3 ± 0.8 ^c^
	5	48.2 ± 1.8 ^ab^	48.0 ± 1.0 ^ab^	47.4 ± 0.6 ^ab^	47.1 ± 0.7 ^b^	48.4 ± 1.2 ^a^
ALH (μm)	1	7.1 ± 0.4 ^bc^	6.8 ± 0.2 ^c^	7.8 ± 0.2 ^a^	7.6 ± 0.3 ^a^	7.1 ± 0.3 ^b^
	2	6.5 ± 0.4 ^d^	6.6 ± 0.2 ^d^	7.5 ± 0.2 ^b^	7.9 ± 0.4 ^a^	7.1 ± 0.3 ^c^
	3	5.7 ± 0.4 ^d^	6.3 ± 0.4 ^c^	7.4 ± 0.4 ^a^	7.5 ± 0.4 ^a^	6.8 ± 0.3 ^b^
	4	6.6 ± 0.4 ^c^	6.3 ± 0.3 ^d^	7.4 ± 0.3 ^a^	7.6 ± 0.3 ^a^	7.1 ± 0.3 ^b^
	5	7.1 ± 0.3 ^c d^	7.3 ± 0.2 ^bc^	7.5 ± 0.3 ^ab^	7.6 ± 0.3 ^a^	6.9 ± 0.3 ^d^
BCF (Hz)	1	43.0 ± 2.2 ^a^	43.1 ± 1.5 ^a^	42.3 ± 1.6 ^a^	40.5 ± 1.2 ^b^	42.0 ± 2.2 ^ab^
	2	39.5 ± 3.5	37.8 ± 1.9	39.4 ± 1.6	38.3 ± 2.1	38.2 ± 2.1
	3	43.6 ± 1.5 ^a^	38.2 ± 1.5 ^b^	39.8 ± 5.2 ^b^	38.1 ± 3.7 ^b^	37.6 ± 1.3 ^b^
	4	42.0 ± 1.8 ^a^	38.4 ± 2.6 ^b^	38.5 ± 1.9 ^b^	38.4 ± 1.9 ^b^	37.8 ± 3.1 ^b^
	5	41.6 ± 2.7 ^a^	39.4 ± 2.3 ^b^	38.6 ± 1.3 ^b^	38.0 ± 0.8 ^b^	38.4 ± 2.0 ^b^

Statistical analysis was performed using one-way ANOVA followed by Tukey’s post hoc test, n = 3. Data are presented as means ± standard deviation from three replicates, with different letters representing significant differences (*p* < 0.05). VCL, curvilinear velocity; VSL, straight-line velocity; VAP, average path velocity; BCF, beat-cross frequency; ALH, lateral head; STR, straightness (VSL/VAP); LIN, linearity (VSL/VCL); WOB, wobble (VAP/VCL).

## Data Availability

The data presented in this study are available in the article.

## Supplementary Materials

The following supporting information can be downloaded at: https://www.mdpi.com/article/10.3390/antiox14010102/s1, Figure S1. Effect of PQQ added to the freezing extender on post-thaw boar sperm viability and acrosome integrity. Figure S2. Effect of supplementation different concentrations of PQQ to the freezing extender on sperm mitochondrial membrane potential in boar sperm post-thaw. Figure S3. Effects of PQQ added to the freezing extender on post-thaw boar sperm mitochondrial ROS levels. Figure S4. Effects of PQQ added to the freezing extender on post-thaw boar sperm mitochondria-encoded proteins (MT-ND1, MT-ND6). Figure S5. Effects of PQQ added to the freezing extender on post-thaw boar sperm -zona pellucida (ZP) binding capacity. (A) zona pellucida. (B) Control group sperm-zona pellucida complex, (C) 1000 nM PQQ group sperm-zona pellucida complexes. Bars = 50 μm.
