# Supplementary material for: Pyrroloquinoline Quinone Improved Boar Sperm Quality via Maintaining Mitochondrial Function During Cryopreservation
Source: Antioxidants (Basel). 2025 Jan 16;14(1):102. doi: 10.3390/antiox14010102 (PMC11763317; doi:10.3390/antiox14010102)

Figure S1. Effect of PQQ added to the freezing extender on post-thaw boar sperm viability and acrosome integrity.

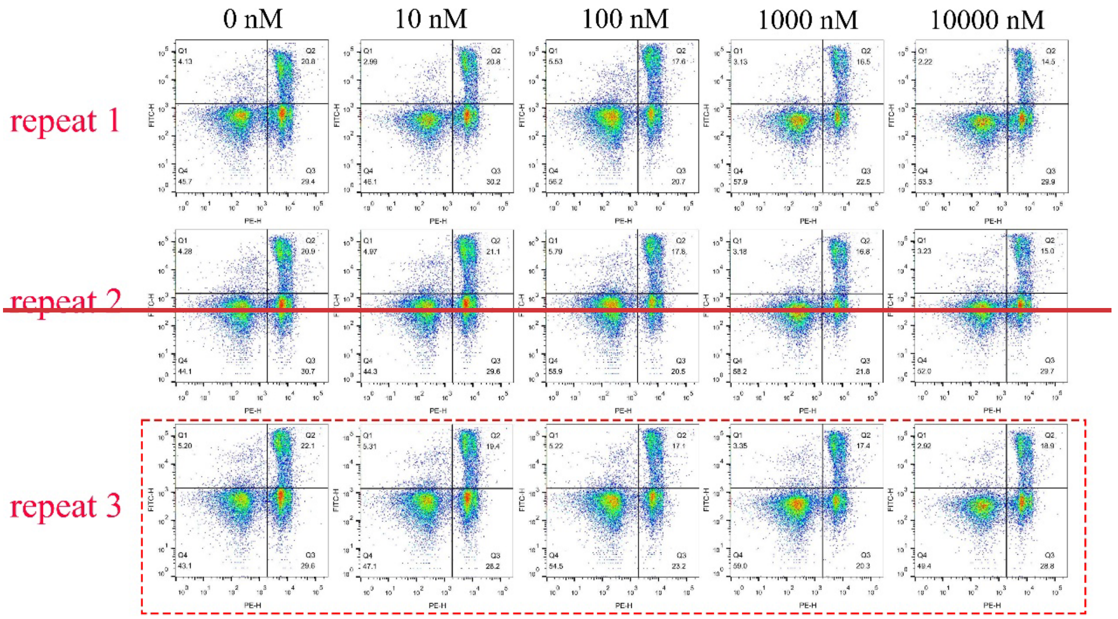

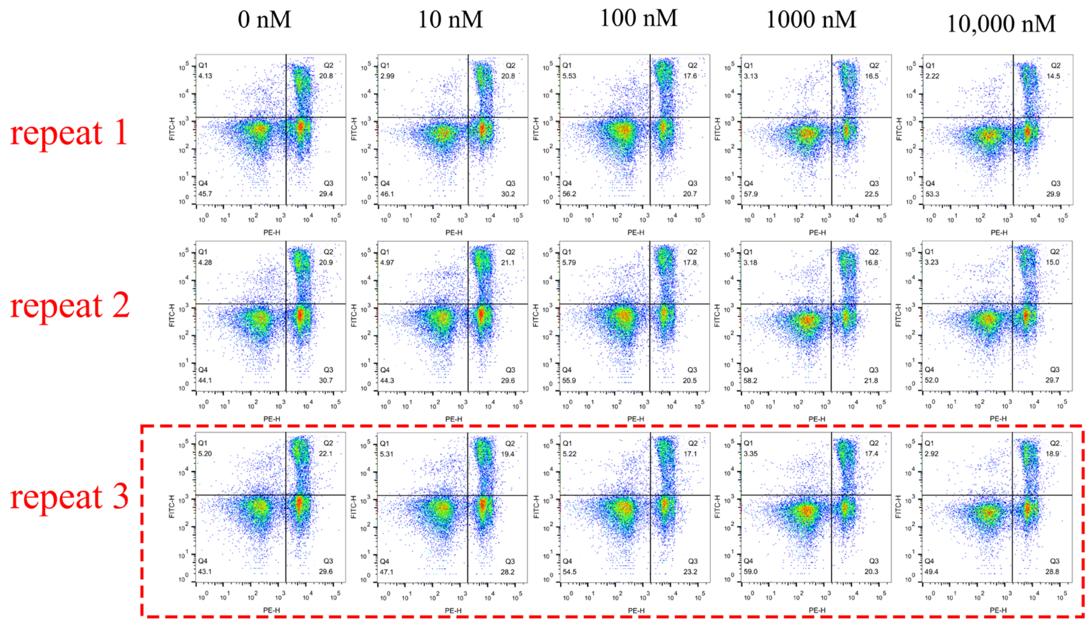

Figure S2. Effect of supplementation different concentrations of PQQ to the freezing extender on sperm mitochondrial membrane potential in boar sperm post-thaw.

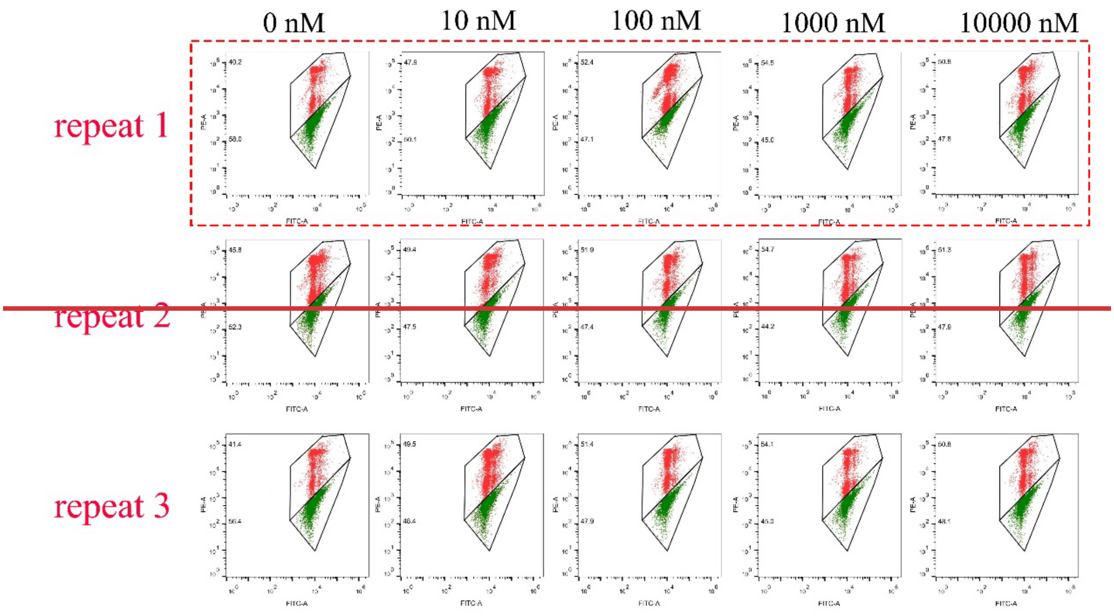

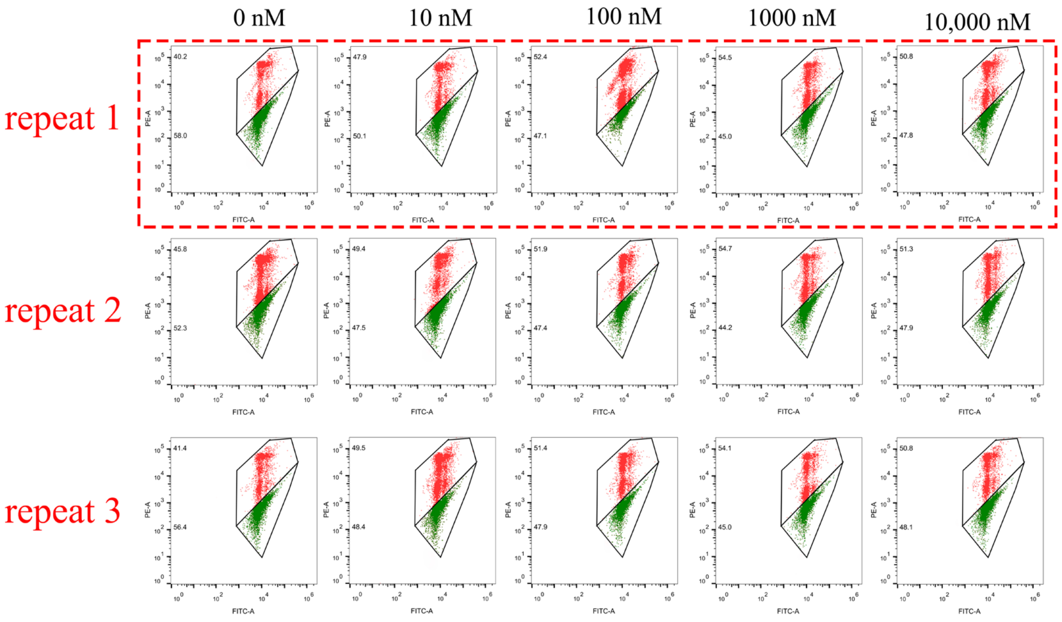

Figure S3. Effects of PQQ added to the freezing extender on post-thaw boar sperm mitochondrial ROS levels.

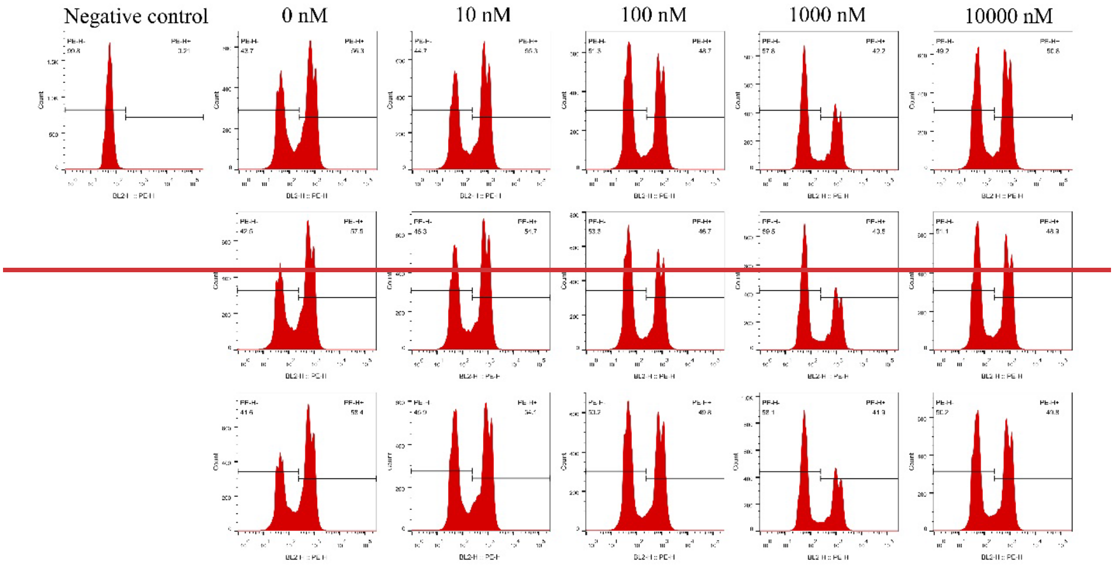

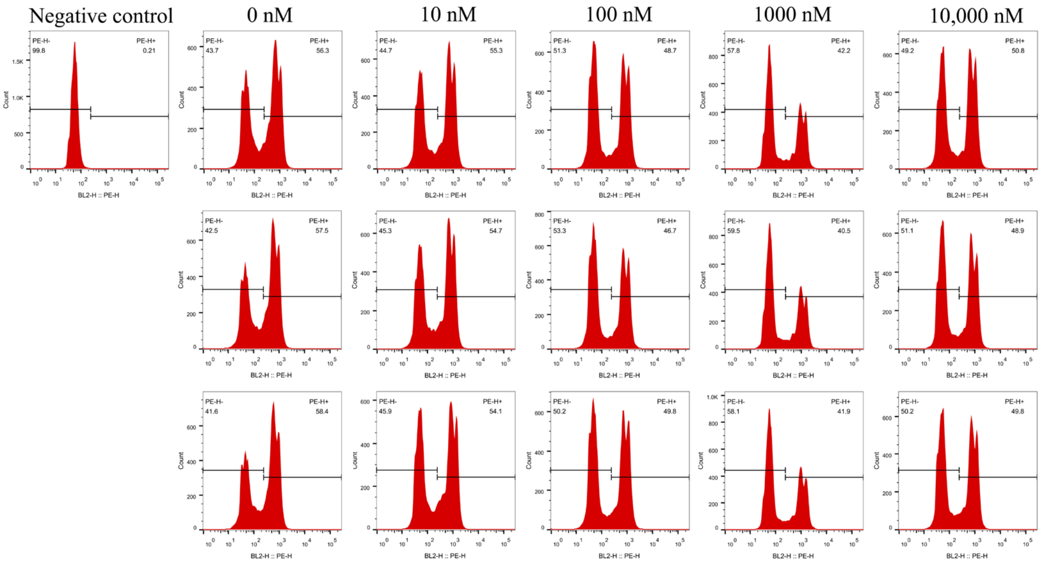

Figure S4. Effects of PQQ added to the freezing extender on post-thaw boar sperm mitochondria-encoded proteins (MT-ND1, MT-ND6).

MT-ND1 Repeat 1

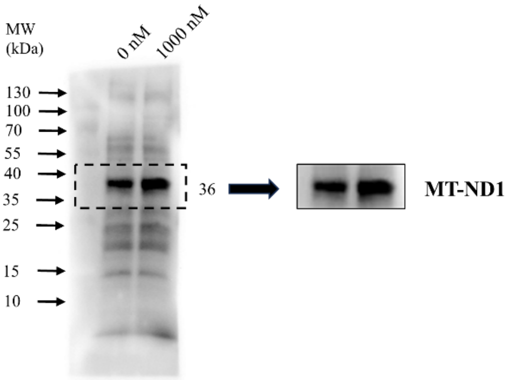

α-Tubulin Repeat 1

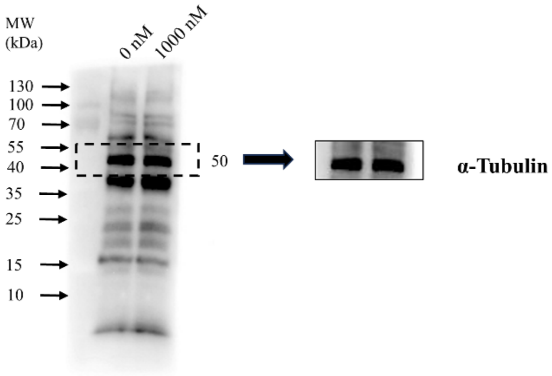

MT-ND1 Repeat 2

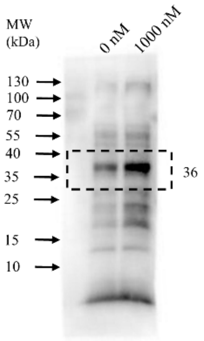

α-Tubulin Repeat 2

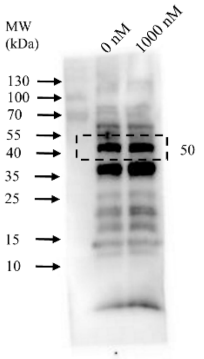

MT-ND1 Repeat 3

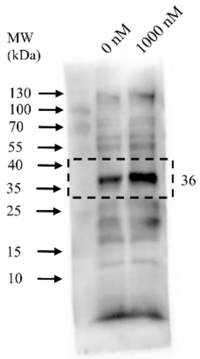

α-Tubulin Repeat 3

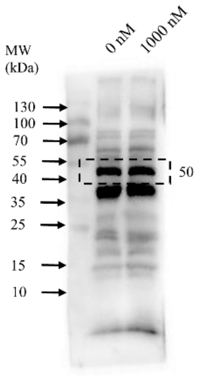

MT-ND6 Repeat 1

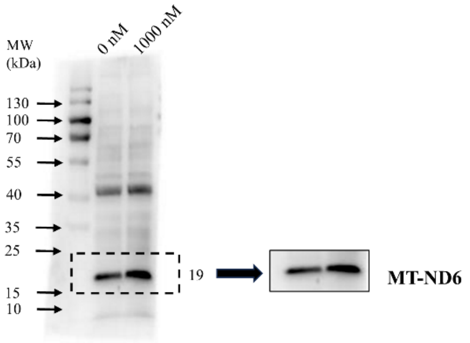

α-Tubulin Repeat 1

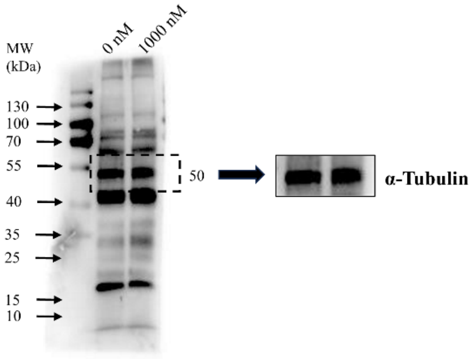

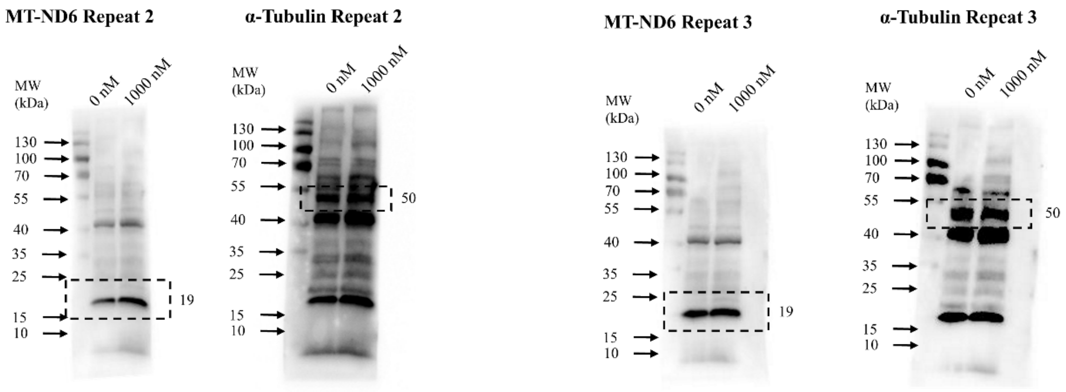

Figure S5. Effects of PQQ added to the freezing extender on post-thaw boar sperm -zona pellucida (ZP) binding capacity. (A) zona pellucida. (B) Control group sperm-zona pellucida complex, (C) 1000 nM PQQ group sperm-zona pellucida complexes. Bars=50 μm.

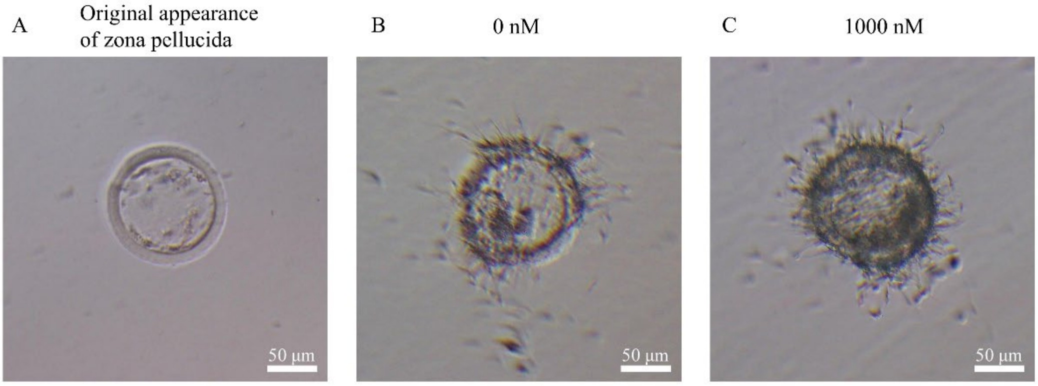

Supplement: Supplementary file 1 [file antioxidants-14-00102-s001.zip › antioxidants-3409014-supplementary.pdf]
